# A systematic review and meta-analysis of the effect of phonophoresis on patients with knee osteoarthritis

**DOI:** 10.1038/s41598-022-16084-8

**Published:** 2022-07-27

**Authors:** Fu-An Yang, Hung-Lun Chen, Chih-Wei Peng, Tsan-Hon Liou, Reuben Escorpizo, Hung-Chou Chen

**Affiliations:** 1 School of Medicine, College of Medicine, Taipei Medical University, Taipei, Taiwan; 2 School of Gerontology Health Management, College of Nursing, Taipei Medical University, Taipei, Taiwan; 3 School of Biomedical Engineering, College of Biomedical Engineering, Taipei Medical University, Taipei, Taiwan; 4 Department of Physical Medicine and Rehabilitation, Shuang Ho Hospital, Taipei Medical University, No. 291 Zhongzheng Road, Zhonghe District, New Taipei City, 235 Taiwan; 5 Department of Physical Medicine and Rehabilitation, School of Medicine, College of Medicine, Taipei Medical University, Taipei, Taiwan; 6 Department of Rehabilitation and Movement Science, College of Nursing and Health Sciences, University of Vermont, Burlington, VT USA; 7 Swiss Paraplegic Research, Nottwil, Switzerland; 8 Center for Evidence-Based Health Care, Shuang Ho Hospital, Taipei Medical University, Taipei, Taiwan

**Keywords:** Health care, Medical research

## Abstract

This systematic review and meta-analysis investigated the effect of phonophoresis when various gel types were used. Medline (using PubMed), EMBASE, and Cochrane Central Register of Controlled Trials (CENTRAL) were used to search for relevant studies from the date of their inception to June 28, 2021. We included studies that were randomized controlled trials (RCTs), included patients with a diagnosis of knee osteoarthritis, included treatment with either phonophoresis or therapeutic ultrasound with placebo gel, and reported clinical and functional outcomes. Continuous variables are expressed as standardized mean differences (SMDs) with 95% confidence intervals (CIs). Statistical analysis was performed using RevMan 5.3 software. We initially retrieved 2176 studies and finally analyzed nine RCTs including 423 patients. The intervention group significantly outperformed the control group in pain scores with NSAID gel (SMD = − 0.53, 95% CI [− 1.02, − 0.05], *I*^2^ = 73%) and in the Western Ontario and McMaster Universities Arthritis Index (WOMAC) function score with corticosteroid gel (SMD = − 0.96, 95% CI [− 1.47, − 0.44], *I*^2^ = 20%). Phonophoresis alleviated pain and improved functional performance. Because of some limitations of this study, additional high-quality, large-scale RCTs are required to confirm the benefits.

## Introduction

Knee osteoarthritis is characterized by breakdown of the articular cartilage over time^1,2^. Although cartilage breakdown is the major disease characteristic, osteoarthritis affects all joint tissues, including the synovial membrane, which is usually associated with increased pain and joint dysfunction^2,3^. Common clinical symptoms include knee pain with gradual onset and that worsens with activity, knee stiffness and swelling, pain after prolonged sitting or resting, and pain that worsens over time^4^. Some studies have reported that approximately 13% of women and 10% of men aged 60 years or older have symptomatic knee osteoarthritis^5,6^.

Treatment initially involves nonsurgical modalities and progresses to surgical treatment once nonsurgical methods are no longer effective^4^. These interventions do not alter the disease process but may substantially reduce pain and disability^7,8^. Self-management programs, muscle strengthening, low-impact aerobic exercises, neuromuscular therapy, and physical activity are recommended for patients with knee osteoarthritis^9–12^. Oral pharmacological agents such as nonsteroidal anti-inflammatory drugs (NSAIDs) and corticosteroids are effective in the treatment of knee osteoarthritis^13–15^. However, oral anti-inflammatory drugs may increase the risks of gastrointestinal, renal, and other systemic toxicities. Topical gels are an alternative treatment option with fewer complications compared with oral anti-inflammatory drugs^16–18^.

Ultrasound, as a treatment modality, has been studied for many decades^19^. Ultrasound exerts a therapeutic effect through the absorption of mechanical energy and the production of heat in tissues^20^. Phonophoresis involves the use of ultrasound to deliver therapeutic drugs by absorption and permeation of the skin^21^. Phonophoresis with an anti-inflammatory gel has been reported to alleviate pain and inflammation in many musculoskeletal conditions^22–25^. Despite the wide use of phonophoresis, scientific evidence supporting its use is insufficient, especially with regard to symptomatic knee osteoarthritis. Wu et al. conducted a systematic review and meta-analysis comparing the effects of therapeutic ultrasound with those of sham ultrasound on knee osteoarthritis^26^. A subgroup analysis indicated that the phonophoresis ultrasound group reported less pain (measured using the visual analog scale [VAS]) than did the conventional nondrug ultrasound group^26^. No significant differences in functional performance (determined on the basis of Western Ontario and McMaster Universities Arthritis Index [WOMAC] score) were observed between the groups^26^. However, only three randomized controlled trials (RCTs) were included in the study to compare the effect of phonophoresis with that of nondrug therapeutic ultrasound. Moreover, according to our electronic database search, more RCTs have been published recently. Thus, this study investigated the effect of phonophoresis for various gel types and compared these effects against those of a placebo (nondrug) gel; these gels were used in therapeutic ultrasound for treating knee osteoarthritis.

## Method

This review was performed in accordance with the recommendations of the Cochrane Handbook for Systematic Reviews of Interventions^27^ and is reported following the guidelines of the Preferred Reporting Items for Systematic Reviews and Meta-Analyses^28^. This systematic review was registered in the International Prospective Register of Systematic Reviews database under the number CRD42021266126 on August 6, 2021.

### Eligibility criteria

We included studies that (1) were RCTs; (2) included patients with a diagnosis of knee osteoarthritis; (3) involved treatment with phonophoresis (with an NSAID, corticosteroid, Chinese herbal gel, or other gel) as the intervention; (4) involved therapeutic ultrasound with a placebo (nondrug) gel as the control treatment; and (5) reported clinical outcomes including pain scores (measured using the VAS) and functional performance (assessed on the basis of WOMAC function score, range of motion, and a walk test). We excluded articles that were protocols, non-peer-reviewed articles, conference papers, and letters to the editor. No language restriction was applied in our search strategy.

### Literature search

We searched electronic databases, namely Medline (using PubMed), EMBASE, and CENTRAL. In our search strategy, we included terms related to both phonophoresis and knee osteoarthritis and their synonyms (the search strategies are presented in the Supplementary Appendix). If available, RCTs were identified using the refined search function of the databases. Additional articles were identified by manually searching the reference lists of relevant articles. The databases were searched from their inception to June 28, 2021.

### Study selection

Only RCTs that compared the effects of phonophoresis with those of nondrug therapeutic ultrasound on pain and physical function in patients with knee osteoarthritis were included. Titles and abstracts were screened to select relevant articles. Two reviewers independently evaluated the eligibility of all titles and abstracts, and disagreements were resolved through discussion. A third reviewer adjudicated any disagreement that could not be resolved through discussion. Subsequently, the full texts of remaining articles were read in detail to determine the eligibility of the articles.

### Data extraction

Two authors extracted data from each study by using a structured form, and the characteristics of all eligible studies are summarized in a table. The following data were extracted: (1) basic information of qualifying studies (first author and publication date); (2) demographic, clinical, and treatment characteristics (e.g., number and mean age of patients in the control and treatment groups); (3) therapeutic ultrasound parameters (mode, frequency, intensity, and duration); (4) content of the gel used in phonophoresis; (5) follow-up period; and (6) outcome measures. Moreover, the means and standard deviations (SDs) of outcome measurements before and after treatment for the experimental and control groups were extracted. If crucial data could not be extracted from an article, we sent an email to the corresponding author, requesting the data.

### Outcome measurements

The outcome measurements of this study were pain score, WOMAC function score, range of motion, walk test score, and adverse events. The pain scores were VAS scores^29^. Higher VAS scores indicate a higher intensity of pain. The WOMAC function score is obtained from a self-administered questionnaire widely used for evaluating hip and knee osteoarthritis^30^. Higher WOMAC function scores indicate higher pain intensity, greater stiffness, and poorer physical function. Range of motion is the range through which a joint can be moved^31^. The walk tests included in this study were the 6-min walk test, timed up and go test, 15-min walk test, and 20-min walk test^32,33^.

### Risk of bias assessment

Risk of bias was examined using the RoB 2 tool, a revision of the Cochrane risk-of-bias tool for RCTs, which is widely used for assessing the quality of RCTs^34^. The following domains were considered: (1) the randomization process, (2) deviations from intended interventions, (3) missing outcome data, (4) outcome measurement, (5) the selection of reported results, and (6) overall bias^34^. Following the Cochrane Handbook for Systematic Reviews of Interventions, the risk of bias was assessed by two independent reviewers^27^. Disagreements between the reviewers were resolved through discussion and consultation with a third reviewer.

### Statistical analysis

Statistical analyses were performed using RevMan 5.3 software, which is provided by the Cochrane Collaboration (https://training.cochrane.org/online-learning/core-software-cochrane-reviews/revman/revman-5-download). Continuous data were extracted as changes from baseline measurements. For studies not reporting SDs, the authors were contacted for raw data or, if unavailable, the data were estimated by calculating correlation coefficients in accordance with the instructions provided in the Cochrane Handbook for Systematic Reviews of Interventions^27^. Results with *P* < 0.05 were considered statistically significant. We used the *I*^2^ test to objectively measure statistical heterogeneity, with *I*^2^ ≥ 75% indicating considerable heterogeneity^35^. A random effects model was used in this meta-analysis due to clinical and methodological heterogeneity. Continuous variables are presented as standardized mean differences (SMDs) with 95% confidence intervals (CIs). The analysis was performed on the basis of different gel contents (corticosteroid, NSAID, herbal gel, or other gel). The meta-analysis was conducted only when at least two RCTs assessed the same gel type. SMDs were used to examine the strength of the relationships between variables in a population; an SMD of < 0.2 was considered to indicate a trivial effect with no clinical meaningfulness; 0.2–0.5 indicated a small effect; 0.5–0.8 indicated a moderate effect; and > 0.8 indicated a large effect^36^.

Sensitivity analysis was performed by excluding one or two studies at a time to examine the stability and reliability of the meta-analysis. We performed this analysis to evaluate the effect of outliers^27^. Articles with a high risk of bias or those reporting dubious results were regarded as outliers. Outliers were identified by the extent to which their removal reduced overall heterogeneity. Furthermore, we identified the probable causes of outliers after performing the sensitivity analysis.

A funnel plot was constructed to examine publication bias if the number of studies included in each analysis was more than 10.

## Results

### Search results

**Figure 1:**
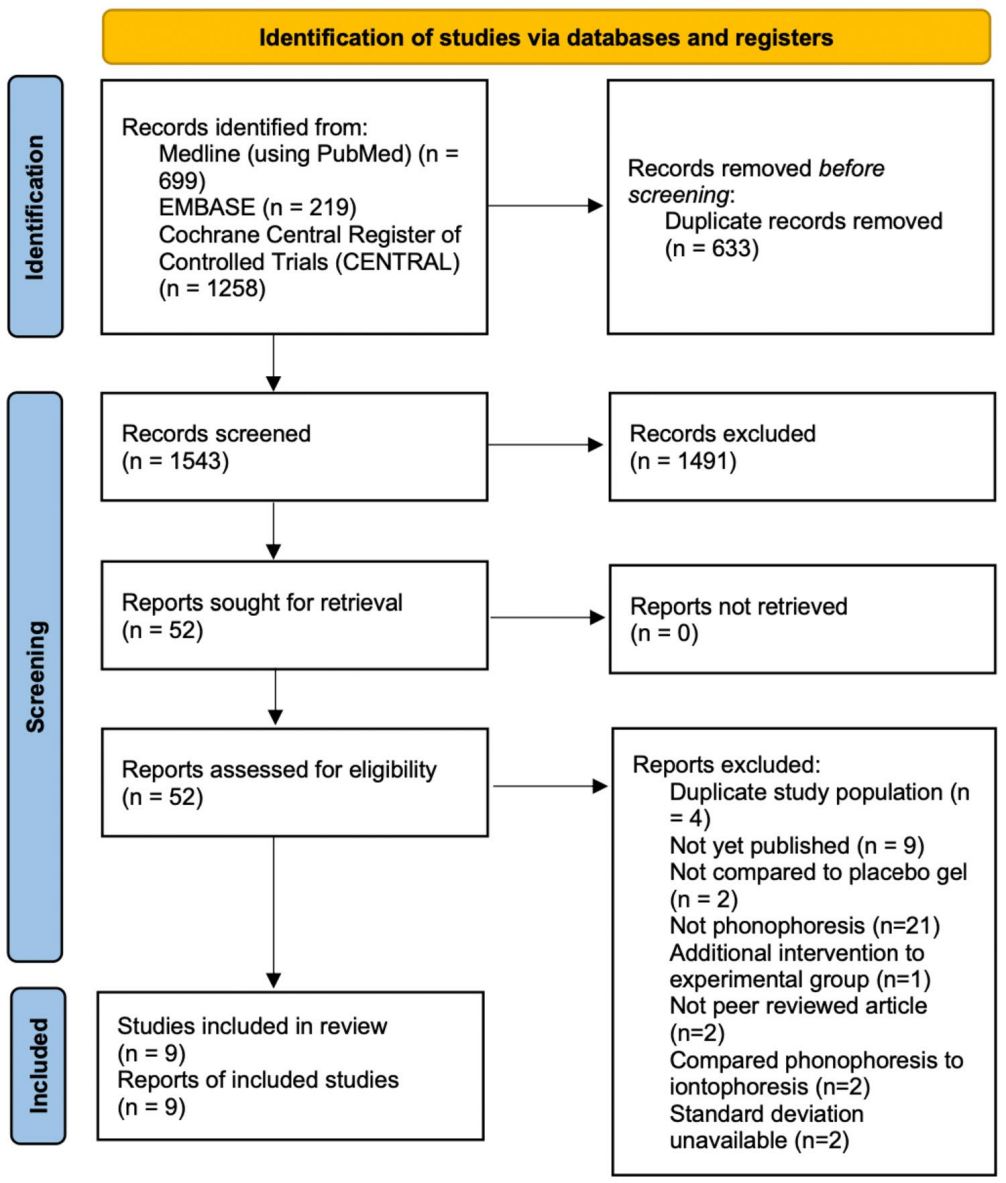
Flowchart of article selection.

By using the search terms mentioned in the supplementary appendix, we initially retrieved 2176 studies. Of these, 633 duplicates were excluded using EndNote X9^37^. Furthermore, 1491 studies that did not meet the inclusion criteria were excluded upon screening of their titles and abstracts. We screened the full texts of the remaining 52 papers and determined that 4 studies included duplicate study populations, 9 were not yet published, 2 did not compare the intervention with placebo gel, 21 did not examine phonophoresis, 1 had an additional intervention to the experimental intervention, 2 were not peer-reviewed articles, 2 compared phonophoresis with iontophoresis, and 2 did not mention SDs. Finally, nine articles were included in the meta-analysis^38–46^. A PRISMA flowchart illustrates the selection process and the number of articles, with reasons included for why studies were excluded at each step of the meta-analysis^47^ (Fig. 1).

### Study characteristics

The selected studies included 222 and 201 patients in the intervention and control groups, respectively. All the selected RCTs were randomized, placebo-controlled trials^38–46^. Two studies used a corticosteroid gel^38,41^, six used an NSAID gel^40,42–46^, and one used a herbal gel^39^. One study had a 1-month follow-up period^40^, and one study had a 3-month follow-up period;^42^ other studies obtained follow-up data within 1 week after the intervention^38,39,41,43–46^. Table 1 lists the main characteristics of the nine RCTs.

**Table 1:** Characteristics of selected randomized controlled trials.

Author, year	Therapeutic ultrasound (mode; frequency; intensity; duration)	Intervention group			Control group			Follow up period	Outcome
		n	Age in years, mean (SD)	Content of gel	n	Age in years, mean (SD)	Content of gel		
Ahmed et al., 2019^38^	Continuous; 1 MHz; 1 W/cm^2^; 10 min	23	53.09 (5.46)	Dexamethasone	23	50.59 (6.77)	Placebo	1 week	Pain score and WOMAC function score
Pinkaew et al., 2019^39^	Continuous; 1 MHz; 1 W/cm^2^; 10 min	20	65.20 (8.34)	*Phyllanthus amarus*	20	64.30 (9.71)	Placebo	1 week	Pain score and 6-min walk test
Zhao et al., 2015^40^	–; 40 kHz; 5000 Pa; –	39	59.4 (8.9)	Diclofenac	19	60.8 (9.0)	Placebo	1 month	Pain score, WOMAC function score, and range of motion
Oktayoğlu et al., 2014^42^	Continuous; 1 MHz; 1.5 W/cm^2^; 10 min	20	54.55 (8.65)	Diclofenac	20	55.05 (10.08)	Placebo	3 months	Pain score and WOMAC function score
Toopchizadeh et al., 2014^41^	–; –; 1.5 W/cm^2^; 5 min	19	54.6 (6.23)	Dexamethasone	18	56.95 (7.33)	Placebo	1 week	Pain score, WOMAC function score, and timed up-and-go test
Boyaci et al., 2013^44^	Continuous; 1 MHz; 1.5 W/cm^2^; 8 min	33	52.45 (4.80)	Ketoprofen	33	52.58 (7.27)	Placebo	1 week	Pain score, WOMAC function score, 15-min walking time
Luksurapan et al., 2013^43^	Continuous; 1 MHz; 1 W/cm^2^; 10 min	23	59.83 (9.88)	Piroxicam	23	58.00 (11.22)	Placebo	1 week	Pain score and WOMAC function score
Akinbo et al., 2011^45^	Continuous; 1 MHz; 1 W/cm^2^; –	15	64.29 (19.83)	Diclofenac	15	64.92 (10.52)	Placebo	1 week	WOMAC function score, range of motion, and 20-min walking time
Kozanoglu et al., 2003^46^	Continuous; 1 MHz; 1 W/cm^2^; 5 min	30	60.3 (9.2)	Ibuprofen	30	59.4 (8.9)	Placebo	1 week	Pain score, WOMAC function score, 20-min walking time, and range of motion

WOMAC, the Western Ontario and McMaster Universities Arthritis Index; SD, standard deviation.

### Risk‑of‑bias assessment

**Figure 2:**
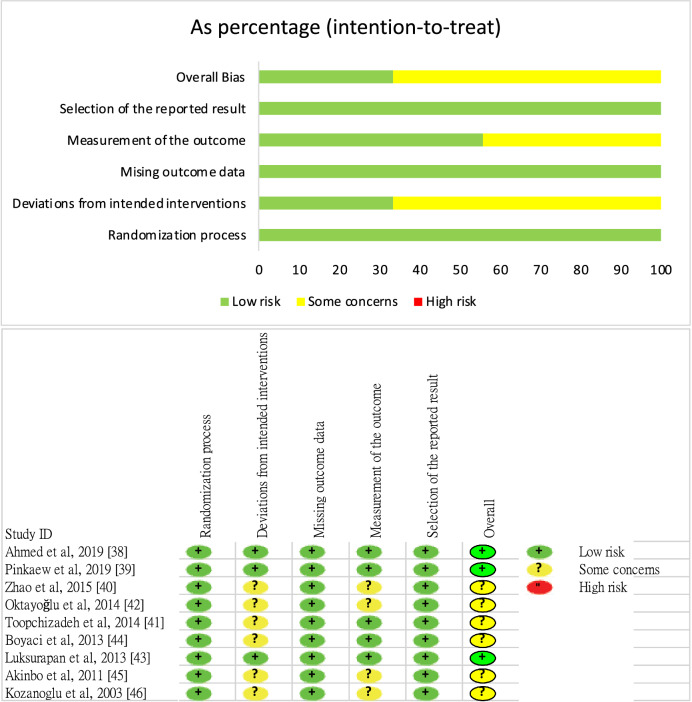
Study quality assessment.

Figure 2 illustrates the risk of bias for each study. Nine studies had low risk associated with the randomization process^38–46^. Six studies exhibited some concerns regarding the risk associated with deviations from the intended intervention^40–42,44–46^, whereas three studies exhibited low risk^38,39,43^. All nine studies had low risk related to missing outcome data^38–46^. Regarding outcome measurements, five studies exhibited low risk^38,39,41,43,44^, and four exhibited some concerns^40,42,45,46^. Regarding the selection of reported results, all nine studies had low risk^38–46^. The overall risk of bias was low for three studies^38,39,43^ and uncertain for six studies^40–42,44–46^.

### Pain scores

**Figure 3:**
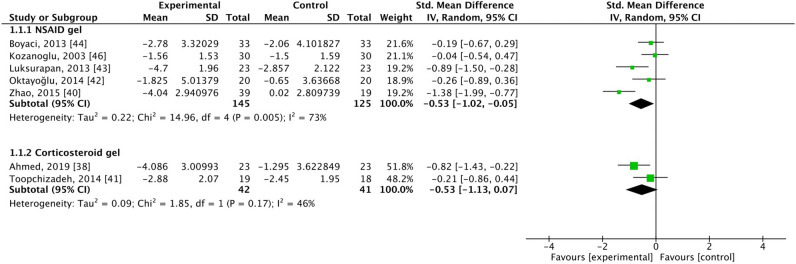
Forest plot for changes from baseline determined using the pain scores. SD, standard deviation; CI, confidence interval.

Pain scores were assessed in five studies where patients were treated with NSAID gel^40,42–44,46^. These studies included 145 patients in the experimental group and 125 patients in the control group. In the analysis, SMD = − 0.53, 95% CI [− 1.02, − 0.05], and *I*^2^ = 73%, which indicated a significant difference and favored the intervention group (Fig. 3).

Additionally, pain scores were assessed in two studies where patients were treated with corticosteroid gel^38,41^. These studies included 42 patients in the experimental group and 41 patients in the control group. In the analysis, SMD = − 0.53, 95% CI [− 1.13, 0.07], and *I*^2^ = 46%, which indicated no significant difference between groups (Fig. 3).

### WOMAC function scores

**Figure 4:**
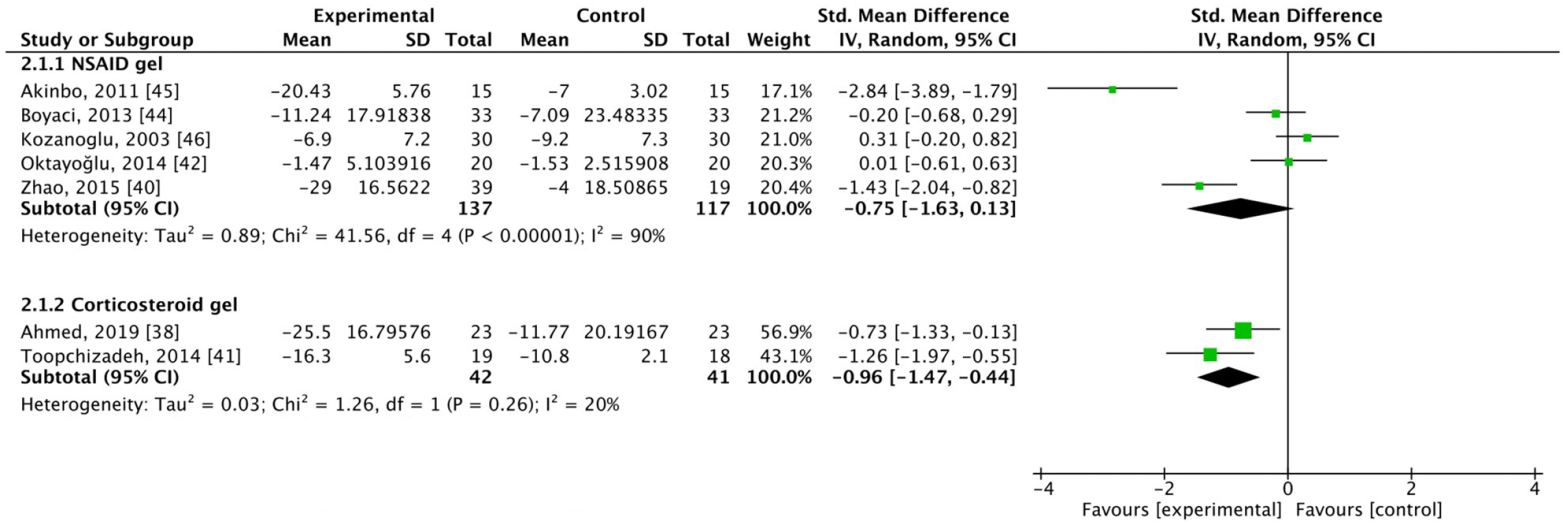
Forest plot for changes from baseline determined using the Western Ontario and McMaster Universities Arthritis Index (WOMAC) function scores. SD, standard deviation; CI, confidence interval.

WOMAC function scores were assessed in five studies where patients were treated with NSAID gel^40,42,44–46^. These studies included 137 patients in the experimental group and 117 patients in the control group. In the analysis, SMD = − 0.75, 95% CI [− 1.63, 0.13], *I*^2^ = 90%, which indicated no significant difference between groups (Fig. 4).

WOMAC function scores were assessed in two studies where patients were treated with corticosteroid gel^38,41^. These studies included 42 patients in the experimental group and 41 patients in the control group. In the analysis, SMD = − 0.96, 95% CI [− 1.47, − 0.44], and *I*^2^ = 20%, which indicated a significant difference and favored the intervention group (Fig. 4).

### Range of motion

**Figure 5:**
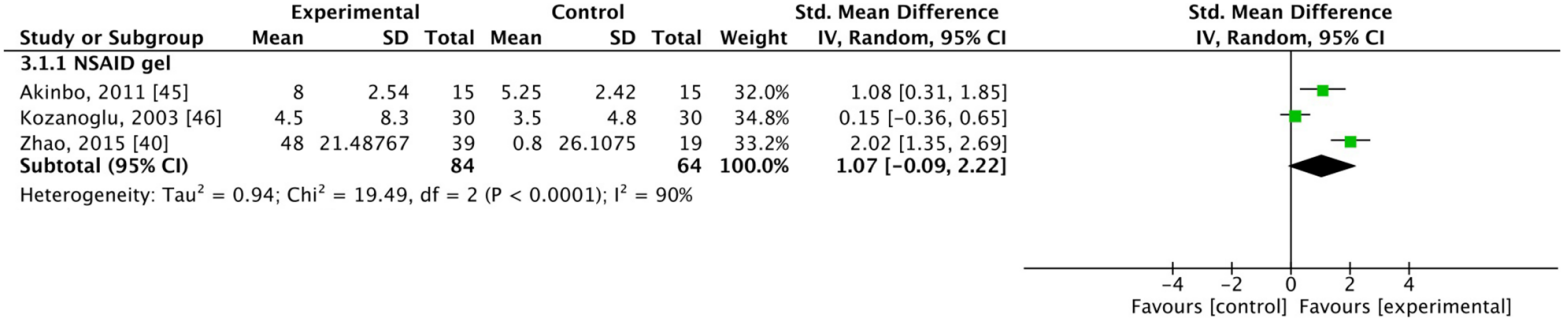
Forest plot for changes from baseline based on range of motion. SD, standard deviation; CI, confidence interval.

The range of motion was assessed in three studies where patients were treated with NSAID gel^40,45,46^. These studies included 84 patients in the experimental group and 64 patients in the control group. In the analysis, SMD = 1.07, 95% CI [− 0.09, 2.00], and *I*^2^ = 90%, which indicated no significant difference between groups (Fig. 5). No study assessed the range of motion when corticosteroid gel was applied.

### Walk tests

**Figure 6:**
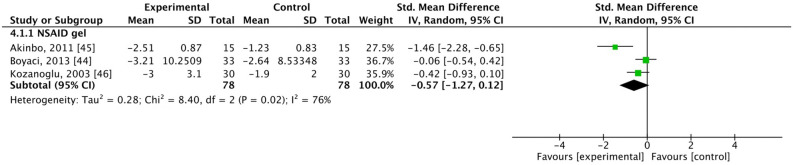
Forest plot for improvement in walk test scores. SD, standard deviation; CI, confidence interval.

Walk tests were adopted in three studies where patients were treated with NSAID gel^44–46^. These studies included 78 patients in the experimental group and 78 patients in the control group. In the analysis, SMD = − 0.57, 95% CI [− 1.27, 0.12], and *I*^2^ = 76%, which indicated no significant difference between groups (Fig. 6).

Only one study evaluated the effect of herbal gel. The study was not included in the meta-analysis because only one RCT was conducted^39^. This study included 20 patients in the experimental group and 20 patients in the control group and focused on pain scores and walk test results. The herbal gel had a larger effect in the intervention group than in the control group in both tests.

### Sensitivity analysis

In the sensitivity analysis, we observed that pain scores with NSAID gel were affected by the removal of studies. However, the effect of NSAID gel on range of motion became significant and favored the intervention group after one article was removed. All results indicated lower heterogeneity after articles were removed (*I*^2^ < 75%; Table 2).

**Table 2:** Sensitivity analysis.

	Content of gel	Outlier(s)	SMD (95% CI) before sensitivity analysis	*I*^2^ before sensitivity analysis	SMD (95% CI) after sensitivity analysis	*I*^2^ after sensitivity analysis
Pain score	NSAID gel	Zhao et al., 2015^40^	− 0.53 (− 1.02, − 0.05)	73%	− 0.32 (− 0.67, 0.03)	38%
	Corticosteroid gel	–	− 0.53 (− 1.13, 0.07)	46%	–	–
WOMAC function score	NSAID gel	Zhao et a.l, 2015^40^ Akinbo et al., 2011^45^	− 0.75 (− 1.63, 0.13)			
	90%	0.04 (− 0.27, 0.35)				
	1%					
	Corticosteroid gel	–	− 0.96 (− 1.47, − 0.44)	20%	–	–
Range of motion	NSAID gel	Kozanoglu et al., 2003^46^	1.07 (− 0.09, 2.22)	90%	1.57 (0.65, 2.49)	69%
	Corticosteroid gel	–	–	–	–	–
Walk test	NSAID gel	Akinbo et al., 2011^45^	− 0.57 (− 1.27, 0.12)	76%	− 0.23 (− 0.58, 0.12)	0%

Significant results are underlined.

WOMAC, the Western Ontario and McMaster Universities Arthritis Index; SMD, standard mean difference; CI, confidence interval.

### Adverse events

Of the nine selected RCTs, four reported adverse effects^39,40,43,46^. No adverse events were observed in these studies, indicating that participants tolerated the interventions well.

## Discussion

Knee osteoarthritis is a degenerative joint cartilage condition^1,2^. The common clinical symptoms of knee osteoarthritis include knee pain that is gradual in onset and worsens with activity, knee stiffness and swelling, pain after prolonged sitting or resting, and pain that worsens over time^4^. Topical anti-inflammatory drugs are an alternative treatment choice with fewer gastrointestinal complications relative to oral drugs^16–18^.

In phonophoresis, ultrasound is used to deliver therapeutic drugs through absorption and permeation of the skin^21^. Despite the wide usage of this treatment, supporting scientific evidence is insufficient, especially with regard to symptomatic knee osteoarthritis. Thus, we conducted this study to investigate the effect of phonophoresis on knee osteoarthritis symptoms. Our analysis revealed significant differences in pain scores that indicated phonophoresis with NSAID gel and significant differences in WOMAC function score that indicated phonophoresis with corticosteroid gel.

We examined the strength of the relationships between variables in a population by determining effect sizes. The results revealed that NSAID gel moderately affected pain scores and that corticosteroid gel greatly affected WOMAC function scores. The findings indicated that phonophoresis could clinically and meaningfully improve the patients’ pain and functional performance. In addition, no study has compared the effects of phonophoresis with those of physical therapy or other types of medical treatments according to our electronic database research. Future studies can fill this research gap.

Therapeutic ultrasound is a deep-heating modality used in physical therapy^42^. According to Rao et al., in therapeutic ultrasound, a transducer converts electrical energy into ultrasound through the piezoelectric principle^48^. Although the exact mechanism underlying its effect remains known, the effect may be composed of two components, namely thermal and nonthermal^49^. In terms of the thermal effect, therapeutic ultrasound induces muscle relaxation and increases connective tissue extensibility and local blood flow, all of which lead to tissue regeneration and reduce inflammation^42,49^. The nonthermal ultrasound effect is related to acoustic cavitation with resultant increases in cell permeability, which is a potential pain relief mechanism^49^. Zhang et al. suggested that therapeutic ultrasound is beneficial for reducing knee pain and improving physical function in patients with knee osteoarthritis and can be a safe treatment option^50^. Phonophoresis refers to the use of ultrasound to deliver therapeutic drugs by absorption and permeation through the skin^21^. The advantage of therapeutic ultrasound is that it may promote the transdermal penetration of therapeutic drugs^43,46^. Moreover, this method is noninvasive and has minimal risk of the adverse effects associated with the systemic administration of anti-inflammatory drugs; it also combines the therapeutic effects of ultrasound and topical drugs^43^. Phonophoresis accounts for up to 30% of physiotherapy visits in some medical centers^45^.

Recently, gels with different contents have been developed for phonophoresis. Corticosteroid and NSAID gels are commonly used. Among the included RCTs, two focused on corticosteroid gels^38,41^, six focused on NSAID gels^40,42–46^, and one focused on herbal gels^39^. The two studies that used corticosteroid gels used dexamethasone gels^38,41^. Among the six RCTs that focused on NSAID gels, three used diclofenac gels^40,42,45^, one used the ibuprofen gel^46^, one used the ketoprofen gel^44^, and one used the piroxicam gel^43^. The herbal gel used was the *Phyllanthus amarus* gel^39^. Although each type of gel exerts anti-inflammatory effects, their chemical properties (e.g., their tissue permeability through ultrasound waves) differ, as reported by Akinbo et al.^45^ In their literature review, Srbely et al. indicated that the depth of penetration of a drug depends on its mass (which is inversely proportional to its molecular weight)^51^. Molecular weight is different from the contents of gels discussed in the selected RCTs. Dexamethasone has high molecular weight; thus, it has a low drug mass and high permeability when applied through ultrasound. The aforementioned reasons may explain why patients in the corticosteroid gel subgroup exhibited greater improvements in some outcomes than did those in the NSAID gel subgroup^45^. Thus, the selection of drug for phonophoresis appears to be as crucial to treatment success as the selection of ultrasound parameters^38^.

Byl et al. reported that diffusion of topically applied drugs through the skin can be enhanced by preheating the skin to increase the kinetic energy^52^. Among our included RCTs, three studies followed this approach^41,45,46^. The application of heat before treatment may have affected the results of these studies. Our analysis indicated that the outcomes reported by the studies that applied preheating were inconsistent. Some studies reported improved outcomes, whereas others indicated no differences when compared with outcomes without preheating application. Therefore, the exact effects of preheating the skin should be investigated in the future.

We conducted a sensitivity analysis by excluding one or two studies at a time to examine the stability and reliability of the meta-analysis. According to the Cochrane Handbook for Systematic Reviews of Interventions, heterogeneity may arise due to the presence of one or two outlying studies with results that are in conflict with those of the remaining studies^27^. If an obvious reason for the outlying result is apparent, the study might be removed with confidence^27^. Both Akinbo et al. and Kozanoglu et al. applied heat to the treatment site before treatment^45,46^. They followed the principle indicated by Byl et al.^52^ However, the application of heat may affect treatment outcomes. The findings of the three studies applying preheating might differ from those of the other included studies^39,45,46^. This difference might explain the change in results in the sensitivity analysis.

In their systematic review and meta-analysis, Wu et al. examined the effectiveness and safety of various therapeutic ultrasound methods^26^. In the subanalysis of phonophoresis, three RCTs were examined^43,44,46^. The results revealed that the phonophoresis ultrasound group had lower pain scores (SMD = − 0.41, 95% CI [− 0.71, − 0.10]) but demonstrated no significant difference in functional performance (assessed on the basis of WOMAC score; SMD = − 0.16, 95% CI [− 0.46, 0.14]). In recent years, more studies on phonophoresis have been conducted^38–46^. We examined the effect of phonophoresis on patients with knee osteoarthritis. We focused on the outcomes of pain and functional performance and determined that phonophoresis effectively improved such outcome measures.

This systematic review and meta-analysis has several strengths. First, this is the first meta-analysis of RCTs investigating the effects of phonophoresis for different types of gels on patients with knee osteoarthritis. Second, our electronic database search indicates that several studies are ongoing in this field. Thus, the results of this study can serve as a reference for future studies. Third, multiple major databases were searched, without language restrictions, prior to the selection of RCTs. Fourth, the data and quality of the selected studies were examined by at least two reviewers through a group consensus approach.

Our study has several limitations that may limit the generalizability of our results. First, heterogeneity was moderate to high for some outcomes, possibly because of varying disease severity, symptom duration, patient characteristics, and treatment protocol. Thus, additional studies are required to establish a standardized treatment protocol. Second, different gel contents, such as lidocaine or capsaicin, that could be applied in the experimental group were not investigated. Furthermore, only one study focused on herbal gels and was not included in our meta-analysis, although it revealed a positive effect. Future studies should examine the effects of different gel contents. Third, some studies did not mention blinding to therapeutics and the blinding of patients or assessors. Hence, some concerns regarding the risk of bias may persist. Fourth, we observed that the pain scores with NSAID gel were affected by the removal of articles when conducting the sensitivity analysis. This might compromise the stability and reliability of the meta-analysis. Fifth, the included studies had short follow-up durations. Therefore, we could not analyze long-term outcomes. Fifth, the sample sizes in each study and the number of studies included for each outcome were different. Thus, the outcomes should be interpreted with caution. Additional high-quality large-scale RCTs with long-term follow-up periods are required to overcome these limitations.

## Conclusion

This is the first meta-analysis of RCTs to investigate and provide adequate evidence for the effect of phonophoresis for different gel types on patients with knee osteoarthritis. Our findings indicated that phonophoresis improves pain and functional performance with a moderate to large effect size over a short-term follow-up with either corticosteroid or NSAID gel. Furthermore, no adverse events were reported in the selected studies. Phonophoresis can be an effective treatment option for patients with knee osteoarthritis. However, because of the limitations of this study, additional high-quality, large-scale RCTs with long follow-up periods are required to confirm the benefit and long-term effects of this intervention.

## Supplementary Information

Supplementary Information.
