# Supplementary material for: A systematic review and meta-analysis of the effect of phonophoresis on patients with knee osteoarthritis
Source: Sci Rep. 2022 Jul 27;12:12877. doi: 10.1038/s41598-022-16084-8 (PMC9329477; doi:10.1038/s41598-022-16084-8)
Supplement: Supplementary file 1 — Supplementary Information. [file 41598_2022_16084_MOESM1_ESM.docx]

**Appendix**

Keywords for the search of different electronic databases

| Database | Search terms for query |
| --- | --- |
| PubMed |  |
| #1 | “sonophoresis” OR “ultrasound” OR ”phonophoresis” |
| #2 | “knee” OR “knees” |
| #3 | “arthropathy” OR “arthritis” OR “arthritides” OR “arthrosis” OR “arthroses” OR “osteoarthrosis” OR “osteoarthroses” OR “osteoarthritides” OR “osteoarthrosis” OR “osteoarthritis” OR “OA” OR “osteoarthritis [MeSH]” |
| #4 | #2 AND #3 |
| #5 | #1 AND #4 |
| #6 | #5 AND “randomized controlled trial” |

| Database | Search terms for query |
| --- | --- |
| Excerpta Medica dataBASE (EMBASE) |  |
| #1 | “sonophoresis” OR “ultrasound” OR ”phonophoresis” |
| #2 | “knee” OR “knees” |
| #3 | “arthropathy” OR “arthritis” OR “arthritides” OR “arthrosis” OR “arthroses” OR “osteoarthrosis” OR “osteoarthroses” OR “osteoarthritides” OR “osteoarthrosis” OR “osteoarthritis” OR “OA” |
| #4 | #2 AND #3 |
| #5 | #1 AND #4 |
| #6 | #5 AND “randomized controlled trial” |

| Database | Search terms for query |
| --- | --- |
| Cochrane library |  |
| #1 | “sonophoresis” OR “ultrasound” OR ”phonophoresis” |
| #2 | “knee” OR “knees” |
| #3 | “arthropathy” OR “arthritis” OR “arthritides” OR “arthrosis” OR “arthroses” OR “osteoarthrosis” OR “osteoarthroses” OR “osteoarthritides” OR “osteoarthrosis” OR “osteoarthritis” OR “OA” |
| #4 | #2 AND #3 |
| #5 | #1 AND #4 |
| #6 | #5 AND “randomized controlled trial” |
